# Supplementary material for: Zebrafish Embryos Display Characteristic Bioelectric Signals during Early Development
Source: Cells. 2022 Nov 12;11(22):3586. doi: 10.3390/cells11223586 (PMC9688842; doi:10.3390/cells11223586)
Supplement: Supplementary file 1 [file cells-11-03586-s001.zip › Supplementary file list.pdf]

Appendix A. Supplementary file list

Figure S1. Unfertilized Egg Vm signaling, ΔF/F, and diagonal pattern of 4-cell stage fish embryo. A–H. Still-frame representative max-projection images from a time-lapse video (Supplementary Video 3-4) of an unfertilized Tg (*ubi*: ASAP1) zebrafish embryo imaged from the animal pole position. The white arrowheads point to random Vm transient spots. **I.** ΔF/F quantifications of ROIs in panels **H**–**N** of Figure 2. **J**. ΔF/F quantifications of ROIs in panels **O**–**U** of Figure 2. All lines in panels **I** and **J** represent the standard change in fluorescence intensity of ROIs for the designated cleavage furrows over time. **K**–**N.** Representative Vm images from a 4-8 cell stage fish embryo showing a different pattern of furrow signaling (the second signal was diagonal from the initial). Scale Bar= 250 μm.

Figure S2. Deep cell Vm transient during the 30% epiboly period. A-F. Time-lapse images of a 30% epiboly gastrula period embryo imaged with a single Z-plane through the center (lateral position). White arrows point to the hyperpolarized cells present within the deep cells. Scale Bar= 250 μm.

Video S1. Light-sheet imaging of Vm signaling during 2-4 cell cleavage stage in Tg (*ubi*: ASAP1). This video shows max projections from the animal pole view of ASAP1 fluorescence localized at the cleavage furrows during positioning, propagation, and deepening processes.

Video S2. Light-sheet imaging of Vm signaling during 4-8 cell cleavage stage in Tg (*ubi*: ASAP1). This video shows ASAP1 fluorescence localized at the cleavage furrows from the animal pole view.

Video S3. Light-sheet imaging of Vm signaling during 8-16 cell cleavage stage in Tg (*ubi*: ASAP1). This video shows ASAP1 fluorescence localized at the cleavage furrows from the animal pole view.

Video S4. Light-sheet imaging of Vm signaling in unfertilized Tg (*ubi*: ASAP1). This video shows ASAP1 fluorescence signals occurring within an unfertilized embryo from the animal pole view.

Video S5. Light-sheet imaging of Vm signaling during early blastula stage in Tg (*ubi*: ASAP1). This video shows ASAP1 transient fluorescent signals occurring over the surface of an embryo from the lateral view.

Video S6. Light-sheet imaging of Vm signaling limited to superficial blastomere during early blastula stage in Tg (*ubi*: ASAP1). This video shows a single z–slice ASAP1 transient fluorescent signals limited to the superficial blastomere margins of an embryo from the lateral view.

Video S7. Light-sheet imaging of intercellular Vm signaling during early blastula stage in Tg (*ubi*: ASAP1). This video shows ASAP1 transient fluorescent signals occurring followed by adjacent signals signaling in seemingly an intercellular correlation.

Video S8. Light-sheet imaging of Vm signaling during gastrula stage in Tg (*ubi*: ASAP1). This video shows Vm transients in the EVL and YSL from the early to late gastrula stage in a dorsal-to-lateral view.

Video S9. Light-sheet imaging of Vm signaling within deeper cell layers during gastrula stage in Tg (*ubi*: ASAP1). This video shows Vm transients occurring within deeper cell layers during mid-gastrulation.

Video S10. Light-sheet imaging of Vm signaling during the early segmentation stage in Tg (*ubi*: ASAP1). This video starts at the end of gastrulation and shows Vm signals becoming stronger in certain tissue regions, such as the somites.

Video S11. Light-sheet imaging of left lateral Vm signaling during somite stage in Tg (*ubi*: ASAP1). This video shows a continuation of Vm transients, dynamic somite hyperpolarizations, and increased signals in the developing heart from the left lateral view.

Video S12. Light-sheet imaging of dorsal Vm signaling during the somite stage in Tg (*ubi*: ASAP1). This video shows a continuation of Vm transients and dynamic unilateral or bilateral, whole and/or partial somite signals from the dorsal view.
