# Supplementary figures and images for: Zebrafish Embryos Display Characteristic Bioelectric Signals during Early Development
Source: Cells. 2022 Nov 12;11(22):3586. doi: 10.3390/cells11223586 (PMC9688842; doi:10.3390/cells11223586)

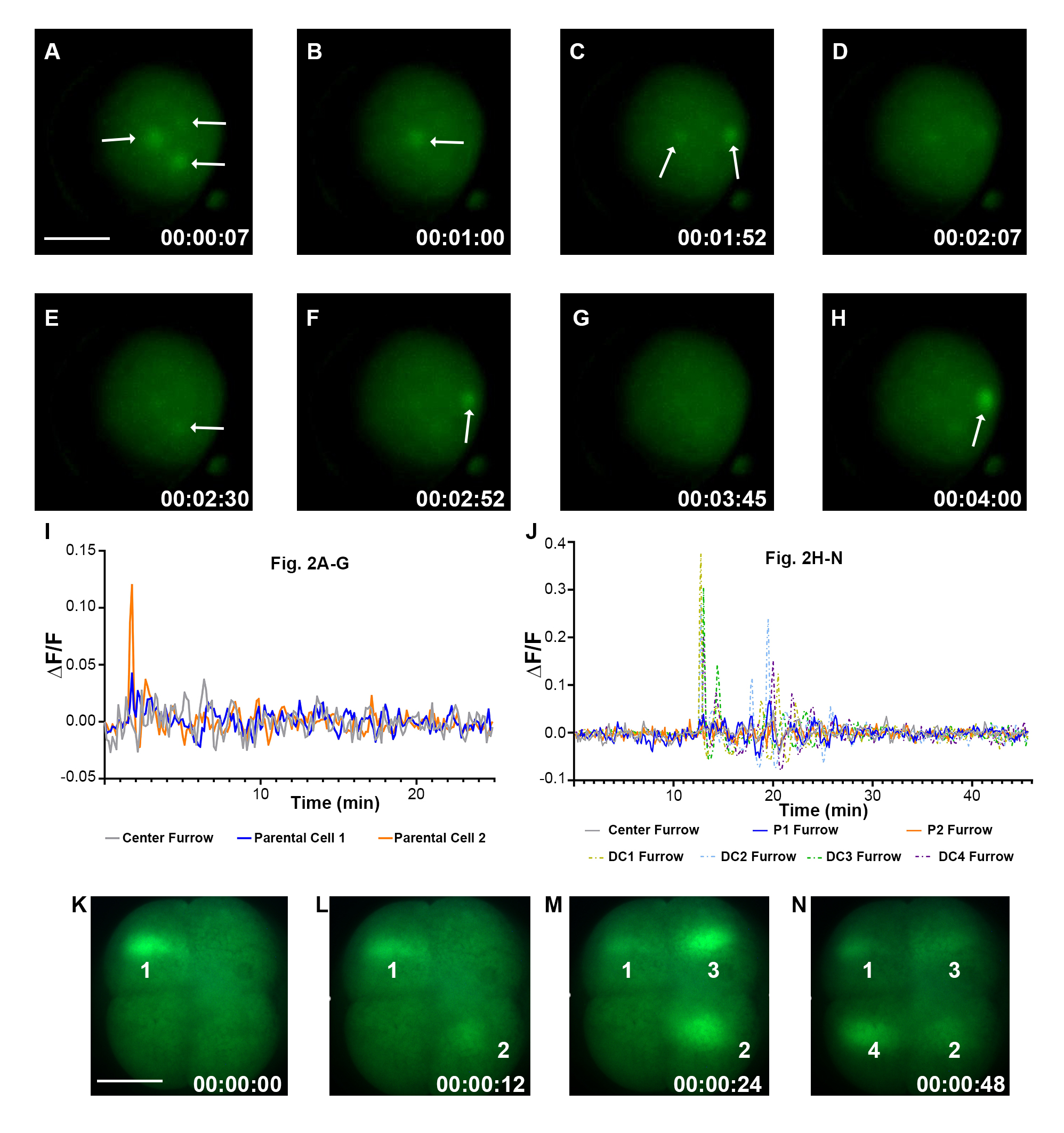

Supplement: Supplementary file 1 [file cells-11-03586-s001.zip › Figure S1.jpg]

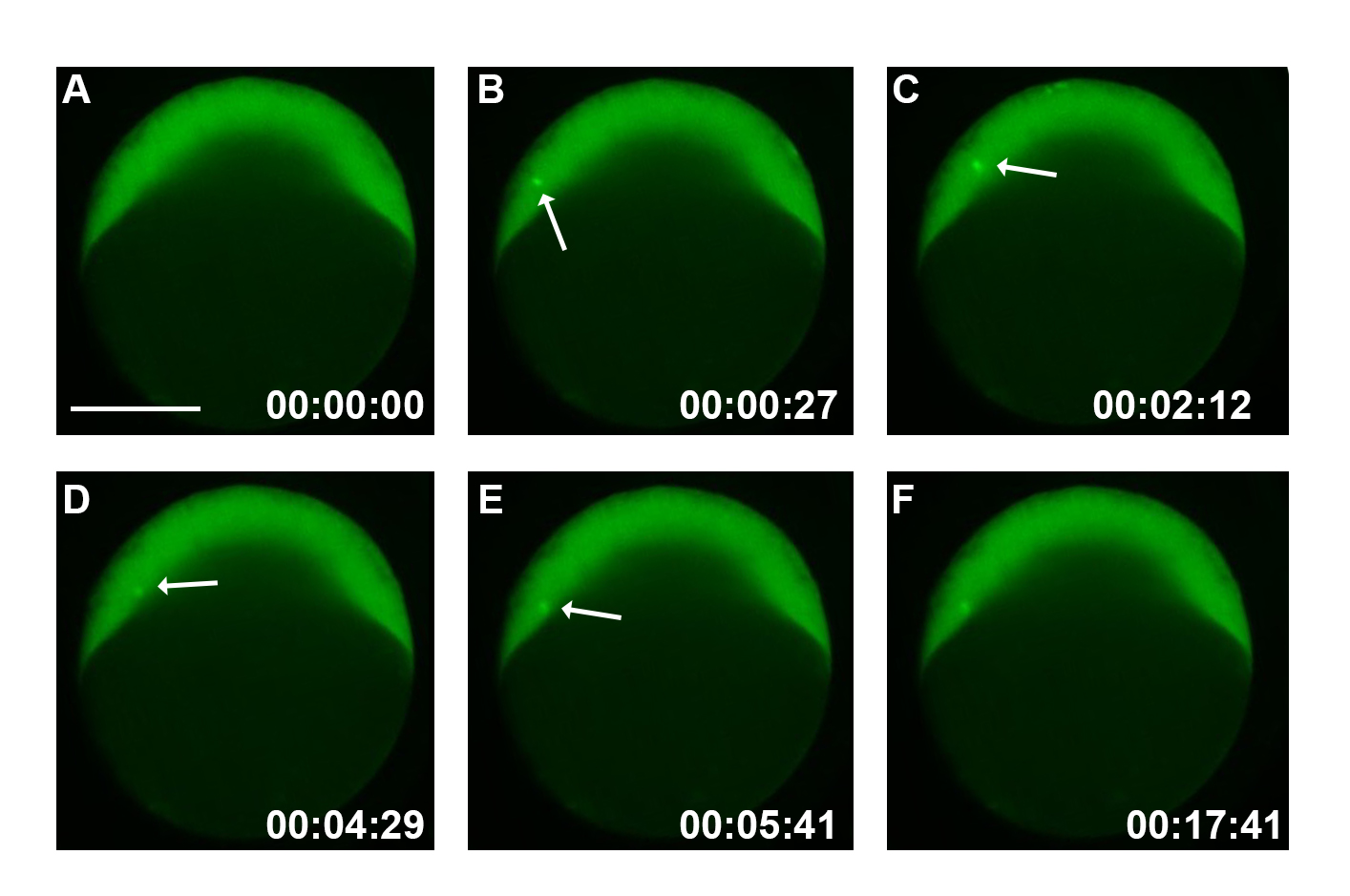

Supplement: Supplementary file 1 [file cells-11-03586-s001.zip › Figure S2.jpg]
